# Beyond usability: how AI tools shape design innovation ability among Chinese industrial design students

**DOI:** 10.3389/fpsyg.2026.1787071

**Published:** 2026-04-13

**Authors:** Xin Wu, Youngcheng Xie, Yunyi Hu, Yu Zeng, Ling Wu, Xinyu Li, Yujie Xiang

**Affiliations:** 1 School of Architecture and Art, Central South University, Changsha, China; 2 School of Information Resource Management, Renmin University of China, Beijing, China; 3 School of Design, Raffles College of Higher Education, Singapore, Singapore; 4 College of Computer Science and Technology, Zhejiang University, Hangzhou, China; 5 School of Creative Studies, Changzhou Vocational Institute of Textile and Garment, Changzhou, China; 6 School of Marxism, Central South University, Changsha, China

**Keywords:** AI tools, design innovation ability, industrial design, information quality, PLS-SEM, technology acceptance

## Abstract

**Introduction:**

With the widespread application of artificial intelligence (AI) technology in design education, exploring how AI tools shape students’ innovative abilities has become increasingly important. Existing technology acceptance models mainly explain the adoption behavior of AI tools but have not examined how technological features influence innovation outcomes through user psychological processes.

**Methods:**

This study employs a cross-sectional quantitative design to examine how AI tool quality dimensions influence design innovation ability (DIA). Specifically, it tests the pathways through which interaction quality (IQT) and information quality (INQ) affect DIA via satisfaction (SAT) and intention to use (INU). Based on an online survey of 1,016 Chinese industrial design students, PLS-SEM data analysis was employed.

**Results:**

The study found that both IQT and INQ positively influence SAT. Subsequently, SAT, IQT, and INQ jointly affect INU. INU significantly predicts DIA. Mediating effect analysis confirmed that SAT plays a partial mediating role between the quality dimensions and INU.

**Discussion:**

Notably, the study reveals a pattern in which information quality exerts stronger effects than interaction quality, and direct functional evaluation outweighs affective mediation, both of which challenge conventional technology acceptance assumptions. The findings extend technology acceptance theory by identifying dual mechanisms, including direct functional and indirect emotional pathways, in AI-assisted creative education.

## 1 Introduction

The integration of artificial intelligence (AI) into design workflows is reshaping creative practice across industries. Contemporary AI-driven platforms, from generative algorithms and machine-learning-enhanced visualization tools to intelligent design assistance systems, have expanded the boundaries of what designers can conceive and produce (Yu et al., 2025; Anantrasirichai and Bull, 2022). In industrial design education, tools such as Midjourney, DALL-E, and Autodesk’s generative design systems are increasingly embedded in studio curricula, enabling students to rapidly explore design alternatives and iterate on concepts (Tsang and Lee, 2022; Chandrasekera et al., 2025). As these tools transition from supplementary aids to integral components of the design process, a critical question emerges: through what mechanisms do AI tools influence students’ development of design innovation ability?

Technology adoption in education has been surveyed in settings such as enterprise resource planning (Soliman and Noorliza, 2023), learning management systems (Li, 2023), and AI-powered chatbots (Al-Adwan et al., 2025), yet AI presents exceptional socio-technical dynamics meriting dedicated theoretical attention. Two theoretical traditions offer particularly relevant lenses for the present context. The Technology Acceptance Model (TAM) identifies perceived usefulness and ease of use as primary determinants of adoption intentions (Davis, 1989; Venkatesh and Bala, 2008). Complementarily, the DeLone and McLean Information Systems Success Model (D&M IS Success Model) posits that system quality, information quality, and service quality drive user satisfaction and subsequent usage behavior (DeLone and McLean, 2002; Petter et al., 2008). These frameworks have been extensively applied and validated in conventional information systems and educational technology contexts (Salisu et al., 2025). However, AI tools present unique socio-technical dynamics that distinguish them from prior educational technologies. Unlike traditional software that executes predefined functions, generative AI tools produce novel content, adapt to individual creative processes, and function as collaborative partners rather than passive instruments (Obrenovic et al., 2025; Heigl, 2025). This fundamental distinction necessitates re-examining whether classical technology acceptance mechanisms operate differently when tools actively participate in creative ideation.

Building on this theoretical foundation, the present study selects four core constructs that capture the distinctive characteristics of AI-assisted creative education. Interaction quality (IQT) and information quality (INQ) are drawn from the D&M model’s quality dimensions, operationalized to reflect the AI-specific attributes of real-time collaborative feedback and generative content quality, respectively. Unlike the generic “system quality” construct in traditional IS research, IQT specifically captures the bidirectional, adaptive nature of human-AI interaction in design contexts, while INQ addresses the accuracy, relevance, and diversity of AI-generated design outputs, dimensions that are uniquely salient when tools produce creative content rather than merely retrieve information. Satisfaction with tools (SAT) represents the affective evaluation pathway, capturing users’ holistic emotional response to the AI tool experience (Hassenzahl and Tractinsky, 2006). Intention to use (INU), by contrast, represents the cognitive evaluation pathway, reflecting deliberate, outcome-oriented decisions about tool adoption. Together, these two constructs form a dual-pathway structure—affective (via SAT) and cognitive (via INU)—that is theoretically grounded in the distinction between hedonic and utilitarian processing in technology acceptance (Han et al., 2024; Nafees and Sujood, 2025). However, this dual-pathway structure has rarely been tested in creative education contexts, where outcome orientation may systematically favor cognitive over affective pathways.

Despite the relevance of these constructs, three interrelated gaps remain in the literature. First, existing studies have not empirically examined the *relative importance* of different AI tool quality dimensions in creative education. Conventional TAM-based guidance assumes that ease of use (analogous to interaction quality) is the primary driver of early adoption, leading educators to prioritize interface-friendly tools and developers to invest in user experience refinement. Yet AI tools in design contexts may derive their value primarily from the quality of generated content rather than interaction fluency. If information quality actually dominates, conventional wisdom misguides resource allocation in both tool development and curriculum design.

Second, the role of affective versus cognitive pathways in AI tool acceptance for creative education remains empirically unresolved. In conventional contexts, satisfaction operates as a key mediator translating quality perceptions into usage intentions. However, design students are trained to evaluate tools based on functional contributions to creative outcomes rather than on emotional responses (Birdi et al., 2016). Whether satisfaction mediates AI tool acceptance to the same degree as in conventional contexts, or whether direct functional evaluation pathways dominate, has significant implications for pedagogical interventions.

Third, existing technology acceptance frameworks treat adoption as the primary outcome, yet in educational contexts, the ultimate goal is ability development (Fan and Jiang, 2024; Sreenivasan and Suresh, 2024). How intention to use relates to design innovation ability development, and what proportion of ability variance technology acceptance constructs can explain, remains underexplored. Without this knowledge, educators cannot determine whether promoting AI tool adoption is sufficient for ability enhancement or whether additional pedagogical scaffolding is required.

These gaps are particularly consequential in the Chinese higher education context. China has the world’s largest population of industrial design students, with over 300 universities offering design programs that have rapidly integrated AI tools into their curricula (Zhai et al., 2021; Zhang et al., 2025). The Chinese educational context presents distinctive characteristics that may moderate AI tool acceptance mechanisms. The emphasis on structured skill acquisition and examination-oriented assessment in Chinese higher education (Zhan et al., 2022) may amplify the cognitive-evaluative pathway, as students are accustomed to assessing tools based on their instrumental value for academic outcomes rather than hedonic satisfaction. Furthermore, the rapid policy-driven adoption of AI in Chinese education, exemplified by the Ministry of Education’s initiatives on AI integration, creates an institutional environment where students encounter AI tools not as optional supplements but as expected components of professional preparation. Understanding AI tool acceptance mechanisms in this specific context is therefore both theoretically informative and practically urgent for the design of curricula, teaching strategies, and AI tool features that effectively support design innovation ability development.

To address these gaps, the present study constructs an integrated model that extends beyond conventional technology acceptance to examine how AI tool quality dimensions influence design innovation ability through user psychological processes. Based on survey data from 1,016 Chinese industrial design students with AI tool experience, we employ Partial Least Squares Structural Equation Modeling (PLS-SEM) to test the model and compare competing pathways. Specifically, the study addresses two research questions:

*RQ1: Through which mechanisms do the interaction quality and information quality of AI tools affect user satisfaction and intention to use?*

*RQ2: How does intention to use impact design innovation ability?*

This study contributes to the literature in three domains. Theoretically, it extends technology acceptance research into AI-assisted creative education by testing whether established TAM and D&M mechanisms hold when tools function as generative collaborators, and by quantifying the relative strength of affective versus cognitive acceptance pathways, an understudied comparison in outcome-oriented learning contexts. Practically, the findings inform curriculum design by revealing which AI tool features most strongly predict design innovation ability development, enabling educators to structure assignments and tool selection criteria that maximize creative learning outcomes. For society, as AI reshapes creative professions globally, understanding how educational technology acceptance translates into professional ability development provides evidence-based guidance for national AI education policies and industry workforce preparation strategies.

The remainder of this paper is organized as follows. Section 2 reviews the relevant literature on AI tools in design education, technology acceptance in AI contexts, and the theoretical foundations underpinning our integrated model, culminating in the development of research hypotheses. Section 3 describes the research methodology, including research design, participants, measurement instruments, and analytical procedures. Section 4 presents the results of the PLS-SEM analysis, including measurement model evaluation, structural model testing, and mediation analysis. Section 5 discusses the findings in relation to existing theory and empirical evidence, and articulates theoretical and practical implications. Section 6 concludes with a summary of key contributions, followed by Section 7 which addresses limitations and directions for future research.

## 2 Literature review and hypotheses development

### 2.1 AI tools in design education

The application of AI tools in design education has evolved rapidly over the past decade. Early implementations were limited to algorithmic generative design and parametric modeling, where AI primarily served as an optimization engine that automated repetitive design calculations (Verganti et al., 2020). More recently, advances in generative AI have fundamentally expanded the scope of AI’s role in design studios. Tools such as Midjourney, DALL⋅E, and Stable Diffusion now enable students to generate visual concepts from textual prompts, explore diverse aesthetic directions within minutes, and iterate on design alternatives at a pace previously unattainable through manual methods alone (Chandrasekera et al., 2025; Tsang and Lee, 2022). This shift has transformed AI from a back-end computational aid into a front-end creative collaborator that actively participates in ideation and concept development.

Empirical research on AI tools in design education has addressed multiple dimensions of this transformation. Studies examining tool integration in studio-based curricula report that AI-generated visual stimuli can broaden the range of concepts students consider during early ideation phases (Yu et al., 2025; Hwang and Wu, 2025). Research on student perceptions indicates generally positive attitudes toward AI tools, with students valuing the speed of concept generation and the ability to visualize abstract ideas rapidly (Anantrasirichai and Bull, 2022). In the Chinese educational context specifically, the scale of AI tool adoption has been substantial: policy initiatives by the Ministry of Education have accelerated the embedding of AI capabilities across design programs, and studies report that Chinese design students increasingly regard AI tools as essential components of their professional toolkit (Zhai et al., 2021; Zhang et al., 2025).

However, the literature on AI tools in design education reveals several critical limitations. First, existing studies are predominantly descriptive, documenting what tools are used and how students perceive them, without systematically examining the mechanisms through which tool characteristics influence educational outcomes. Second, there is a notable absence of theoretically grounded frameworks linking AI tool features to creativity or design innovation ability development. Most studies treat AI tools as a monolithic category, failing to differentiate which specific quality dimensions, interaction quality, content quality, or other attributes, most strongly predict beneficial outcomes. Third, while a growing body of evidence addresses AI in design education globally, comparative analyses between Western and Chinese educational contexts remain scarce, despite significant differences in pedagogical traditions, assessment cultures, and institutional structures that may moderate AI tool acceptance mechanisms (Zhan et al., 2022). These limitations collectively point to the need for theory-driven empirical research that moves beyond descriptive accounts to examine causal pathways from AI tool quality characteristics to design innovation ability outcomes.

### 2.2 Technology acceptance in AI contexts

Research on technology acceptance in educational settings has generated a substantial evidence base, initially grounded in the Technology Acceptance Model (TAM) and subsequently extended through frameworks such as the Unified Theory of Acceptance and Use of Technology (UTAUT) and the DeLone and McLean Information Systems Success Model (D&M IS Success Model). TAM-based studies have consistently demonstrated that perceived usefulness and perceived ease of use predict adoption intentions for educational technologies ranging from learning management systems to mobile learning platforms (Davis, 1989; Venkatesh and Bala, 2008; Salisu et al., 2025). More recent investigations have extended TAM to AI-specific contexts, finding that constructs such as AI trust, perceived intelligence, and algorithmic transparency emerge as additional predictors beyond traditional TAM variables (Soliman et al., 2025). For instance, Soliman et al. (2024) modeled continuous intention to use generative AI among university students using PLS-SEM and artificial neural networks, revealing that performance expectancy and hedonic motivation were the strongest predictors, findings that underscore the need to examine both utilitarian and hedonic pathways in AI acceptance research.

The D&M IS Success Model has provided a complementary perspective by foregrounding quality dimensions, system quality, information quality, and service quality, as antecedents of user satisfaction and system use (DeLone and McLean, 2002; Petter et al., 2008). Studies applying the D&M framework in educational technology contexts have generally confirmed that information quality is a strong predictor of user satisfaction, often surpassing system quality in explanatory power (Luo et al., 2025). However, empirical findings are not entirely consistent. Some studies report that system quality (analogous to interaction quality) dominates satisfaction formation in user-centric applications (Li, 2023), while others find information quality to be the primary driver in content-generation contexts (Ahmad et al., 2025). This inconsistency suggests that the relative importance of quality dimensions may be context-dependent, varying according to whether the technology’s primary function is process facilitation or content generation, a distinction that is particularly relevant for generative AI tools.

Despite these advances, the inconsistencies reviewed above converge on the three research gaps identified in the Introduction: (i) the absence of simultaneous testing of interaction quality and information quality within a single model in creative education contexts, which prevents comparison of their relative predictive strength; (ii) the untested assumption that satisfaction-mediated affective pathways operate equivalently in outcome-oriented educational contexts as in consumer settings; and (iii) the lack of empirical extension from technology adoption to ability development outcomes. Addressing these gaps requires an integrated theoretical framework that combines the quality-driven mechanisms of the D&M model with the acceptance logic of TAM, while extending the outcome variable beyond adoption to ability development. The following section develops this integrated theoretical foundation.

### 2.3 Theoretical foundation

The present study integrates three complementary theoretical perspectives to construct its analytical framework: the Technology Acceptance Model (TAM), the DeLone and McLean Information Systems Success Model (D&M IS Success Model), and User Experience Theory. Before elaborating on this integration, it is necessary to justify why these specific frameworks were selected over alternative theories that have been applied in technology adoption research.

Several established theories were considered but deemed less suitable for the present research objectives. The Theory of Planned Behavior (TPB) (Ajzen, 1991) incorporates subjective norms and perceived behavioral control as predictors of behavioral intention, but its emphasis on social influence is less directly relevant in individual-tool interaction contexts where students independently engage with AI design tools. The Unified Theory of Acceptance and Use of Technology (UTAUT) (Venkatesh et al., 2003) offers a comprehensive multi-predictor framework, but its breadth comes at the cost of parsimony, and its facilitating conditions construct overlaps substantially with the quality dimensions already captured by the D&M model. The Diffusion of Innovation (DOI) theory (Rogers, 2003) focuses on adoption dynamics at the population level, whereas the present study examines individual-level psychological mechanisms linking tool quality to innovation outcomes. The Theory of Reasoned Action (TRA) (Fishbein and Ajzen, 1975), as a precursor to TPB, shares the same limitations regarding social influence constructs in individual tool-use contexts.

TAM provides the core logic of technology acceptance: perceived usefulness and perceived ease of use shape behavioral intentions, which in turn predict actual use (Davis, 1989; Venkatesh and Bala, 2008). TAM has been extensively validated across educational technology contexts (Zhai et al., 2021; Soliman et al., 2025) and offers a parsimonious framework for understanding how functional perceptions drive adoption decisions. However, TAM alone does not specify the quality antecedents that shape perceived usefulness, nor does it differentiate between affective and cognitive evaluation pathways, limitations that are particularly consequential when studying generative AI tools that simultaneously affect users through interaction processes and content outputs.

The D&M IS Success Model addresses these limitations by specifying quality dimensions, system quality, information quality, and service quality, as antecedents of user satisfaction and usage behavior (DeLone and McLean, 2002; Petter et al., 2008). The model’s explicit inclusion of satisfaction as a mediating construct provides the theoretical basis for distinguishing affective evaluation (via satisfaction) from cognitive evaluation (via direct quality-to-intention pathways). In AI tool research, information quality is especially relevant because generative AI systems produce novel content to support user tasks, making content quality a salient evaluation criterion that is distinct from interface usability (Luo et al., 2025).

User Experience Theory contributes the perspective that effective human-technology interaction involves bidirectional processes of cognitive adaptation, emotional resonance, and behavioral support (Hassenzahl and Tractinsky, 2006; Reig et al., 2022). This theory provides the conceptual foundation for operationalizing interaction quality as an independent construct, distinct from the D&M model’s system quality. In AI-assisted design contexts, interaction quality captures the responsiveness, adaptability, and collaborative intelligence of the tool, attributes that reflect AI’s distinctive capacity to function as an intelligent collaborator rather than a passive processor (Stige et al., 2024; Obrenovic et al., 2025).

The integration rationale. Each framework alone addresses only part of the phenomenon under investigation. TAM explains acceptance decisions but not their quality antecedents or educational outcomes. The D&M model specifies quality-to-satisfaction-to-use pathways but does not extend to ability development outcomes. User Experience Theory captures the interactive nature of AI tools but lacks the structural model for hypothesis testing. By integrating these three perspectives, the present study constructs a two-dimensional quality framework based on interaction quality and information quality. These quality dimensions feed through dual evaluation pathways—affective (via satisfaction) and cognitive (via direct intention)—to ultimately predict design innovation ability, thereby connecting technology acceptance research to educational outcome research. This integration is not merely additive; it generates testable propositions that cannot be derived from any single framework alone, specifically: (a) the relative predictive strength of IQT versus INQ in creative education, (b) the mediating versus direct pathway comparison, and (c) the adoption-to-ability link.

### 2.4 Hypotheses development

#### 2.4.1 Interaction quality

Interaction quality (IQT) refers to the extent to which AI tools facilitate effective human-computer collaboration. This concept is derived from the “interaction adaptability” construct in User Experience Theory. In AI-assisted design contexts, interaction quality has three core dimensions: cognitive-level intelligent understanding and feedback, emotional-level personalized experiences, and social-level collaborative support (Obrenovic et al., 2025). This multi-level interaction framework reflects the composite nature of AI tools, which are not only technological instruments but also cognitive partners and collaborative mediums.

When AI tools accurately understand the user’s intentions, provide appropriate feedback, and support effective collaboration, the user’s actual experience is likely to meet or exceed expectations, resulting in higher satisfaction (da Silva et al., 2022). This response reflects both the functional and emotional value derived from the tool. In design practice, a good interaction experience can reduce cognitive load, enhance work efficiency, and increase overall user satisfaction with the tool (Kosch et al., 2023).

High-quality human-computer interaction can create an immersive experience, stimulating intrinsic motivation and exploratory interest in the user (Bahari, 2023). This experience is particularly relevant in creative design contexts, where the creative process is exploratory and uncertain, requiring flexible interactive support from tools (Gao and Huang, 2022). When users perceive that AI tools understand their creative intentions and provide effective collaboration, they develop a strong motivation for continued use (Zhang et al., 2024). Accordingly, we hypothesize:

*H1*: Interaction quality (IQT) of AI tools has a positive impact on tool satisfaction (SAT).

*H2*: Interaction quality (IQT) of AI tools has a positive impact on intention to use (INU).

#### 2.4.2 Information quality

Information quality (INQ), as a core construct in the D&M model, takes on new theoretical significance in the context of AI tools. Traditional information systems focus on the accuracy and completeness of data; however, the evaluation of AI-generated content involves multiple criteria such as creativity, relevance, and usability (Ahmad et al., 2025). In this study, the information quality of AI tools is defined as the extent to which AI-generated content meets users’ design needs, including four core dimensions: accuracy, which reflects the objective correctness of the information; relevance, which indicates how well the information matches the user’s needs; comprehensibility, which refers to the cognitive accessibility of the information; and richness, which reflects the diversity and depth of the information.

High-quality information output directly affects users’ perception of the value of AI tools. When generated content effectively supports design tasks, users experience positive usage outcomes (Luo et al., 2025). This value perception is then transformed into satisfaction through an expectation confirmation mechanism: when AI-generated content exceeds expectations in accuracy, relevance, and usability, users become satisfied. In design practice, high-quality information support enhances creative efficiency and design quality, thus increasing users’ overall satisfaction (Semeraro et al., 2021).

The direct influence of information quality on intention to use reflects the core role of perceived usefulness in the TAM model (Calisir et al., 2014). When users believe AI tools provide high-quality design support, they are motivated to continue using them (Bai and Wang, 2025). In creative work, rich and accurate information broadens the design solution space and inspires novel ideas, increasing willingness to use AI tools (Zhan et al., 2022). Based on this analysis, we propose the following hypotheses:

*H3*: Information quality (INQ) of AI tools has a positive impact on tool satisfaction (SAT).

*H4*: Information quality (INQ) of AI tools has a positive impact on intention to use (INU).

#### 2.4.3 Tool satisfaction

Tool satisfaction (SAT) serves as the affective mechanism through which quality perceptions are translated into behavioral intentions. Having established that both interaction quality and information quality generate satisfaction through process-oriented and outcome-oriented pathways, respectively, the critical question becomes why satisfaction, once formed, drives continued use.

Satisfaction operates as an integrative evaluation: on the cognitive level, it integrates assessments of multiple tool features into an overall value judgment (Zhao et al., 2012); on the emotional level, it captures the subjective affective response to the usage experience (Nafees and Sujood, 2025). This dual nature gives satisfaction its mediating power, as it transforms discrete quality perceptions into a summary appraisal that informs subsequent behavioral decisions (Han and Sa, 2022). In AI tool contexts specifically, satisfaction functions as an emotional anchor that sustains engagement beyond initial trial use, bridging the gap between one-time quality evaluation and continued adoption commitment (Lam, 2025; Li, 2023). This reasoning leads to the following hypothesis:

H5: Tool satisfaction (SAT) has a positive impact on intention to use (INU).

#### 2.4.4 Intention to use and design innovation ability

Intention to use (INU), as a manifestation of behavioral intention, has been applied as a predictor of actual behavior and outcomes in various research fields (Chueh and Huang, 2023). In technology-driven learning contexts, intention to use not only predicts technology adoption behavior but also predicts the depth and continuity of technology integration (Al-Adwan et al., 2025). Behavioral intention is a proximal predictor of actual behavior, and sustained engagement with a technology facilitates skill development.

Design innovation ability (DIA) refers to an individual’s comprehensive ability to identify problems, generate creative solutions, and transform those solutions into feasible designs in the design practice (Birdi et al., 2016). In AI-assisted design environments, this ability develops differently. Users must leverage AI’s generative capabilities while maintaining independent critical thinking and creative judgment (Verganti et al., 2020). Design innovation ability develops through experiential practice and reflective learning, processes that AI tools can meaningfully support by enabling rapid prototyping and iterative feedback.

Users with a strong intention to use are more likely to use AI tools frequently and deeply in their design practice, accumulating experience through repeated human-computer collaboration (Xie et al., 2023). This experience accumulation may occur through multiple pathways, improving tool proficiency, expanding design thinking, and enhancing creative confidence. However, as our study measured design innovation ability as a unified construct rather than testing these pathways separately, these remain theoretical propositions. Future research should examine these pathways empirically. Through continuous AI-assisted design practice, users gradually master effective human-computer collaboration patterns and learn to make full use of AI’s generative capabilities while retaining creative control (Chandrasekera et al., 2025).

Users observe the creative generation process of AI tools, learning new design methods and cognitive strategies. This mechanism aligns with observational learning as conceptualized within social cognitive theory (Bandura, 1986), whereby individuals acquire new behavioral patterns and cognitive strategies through observing models, in this case, the design processes generated by AI tools. Meanwhile, successful collaboration experiences enhance users’ innovative confidence, further promoting the development of design innovation ability. The confidence-building mechanism warrants particular theoretical attention. Self-efficacy theory posits that mastery experiences, successful task completion, constitute the most powerful source of efficacy beliefs, which subsequently influence motivation and performance in similar domains (Usher, 2009). In AI-assisted creative contexts, when students successfully collaborate with AI tools to generate novel design solutions or overcome design challenges, these accomplishments function as mastery experiences that strengthen their creative self-efficacy (Hwang and Wu, 2025). Recent empirical evidence demonstrates that creative self-efficacy significantly predicts innovative behavior and creative performance in design education (Urban and Urban, 2025). Furthermore, AI tool affordances may amplify this confidence-building process by enabling students to explore design possibilities beyond their current skill levels, thereby expanding their perceived creative capabilities (Anantrasirichai and Bull, 2022). This psychological mechanism, where successful AI collaboration enhances confidence, which in turn motivates deeper engagement, creates a positive feedback loop supporting design innovation ability development. Users do not passively accept AI-generated content but actively collaborate with AI, developing design innovation ability (Jin et al., 2025). We therefore hypothesize:

H6: Intention to use (INU) has a positive impact on design innovation ability (DIA).

### 2.5 Research model

Based on the theoretical foundations and hypotheses outlined above, this study identifies key variables in the application of AI tools and their relationships. From the perspective of technological features, interaction quality reflects the ability of AI tools to support human-computer collaboration, while information quality reflects the extent to which AI-generated content meets design needs. From the perspective of user response, satisfaction acts as a mediating variable that translates technology features into behavioral intentions, reflecting the expectation confirmation process in which users compare actual experiences with prior expectations, while intention to use represents a direct precursor of behavior, reflecting the user’s inclination to integrate AI tools into design practice.

**FIGURE 1:**
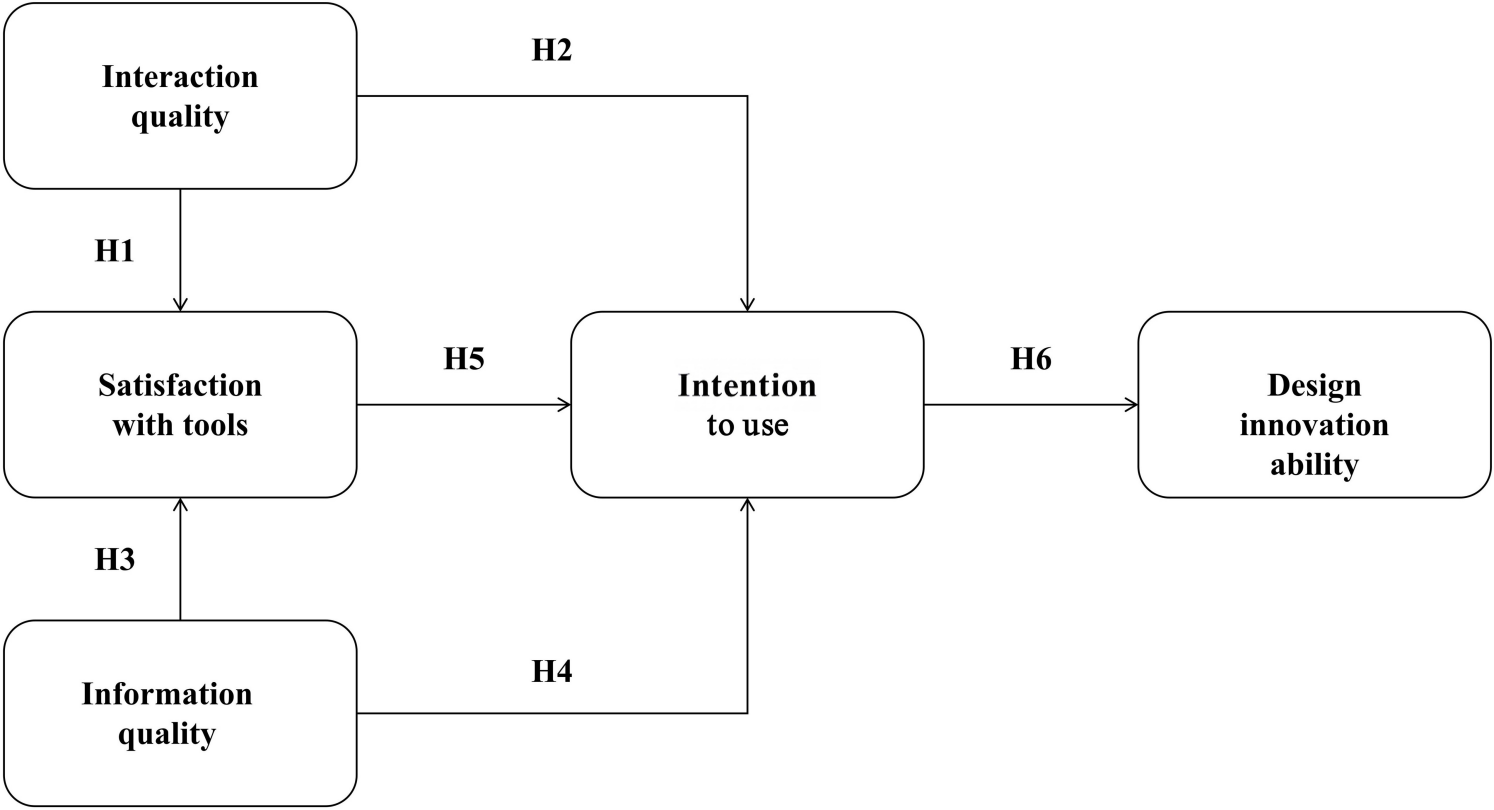
Proposed research model linking AI tool quality to design innovation ability.

Based on this, the study constructs a theoretical model containing five constructs (see Figure 1). The model hypothesizes that interaction quality and information quality affect intention to use through two direct and indirect paths, with satisfaction playing a mediating role. Intention to use, in turn, affects design innovation ability. This model reflects the mechanism by which AI tool features translate into design innovation ability through user cognitive and emotional processes, providing a theoretical framework for exploring the role of AI tools in design practice.

As illustrated in Figure 1, the model follows a three-stage logical flow: AI tool quality characteristics → user psychological responses → innovation ability development. Specifically, information quality (INQ) and interaction quality (IQT) serve as exogenous quality constructs that influence the two user psychological response variables through four direct paths: INQ → SAT (H3), INQ → INU (H4), IQT → SAT (H1), and IQT → INU (H2). Satisfaction (SAT), as an affective mediator reflecting expectation confirmation, further influences intention to use (INU) through the SAT → INU path (H5). Finally, intention to use translates into design innovation ability (DIA) through the INU → DIA path (H6). The model thus captures both direct (cognitive-functional) and indirect (affective-experiential) pathways from perceived AI tool quality to design innovation ability.

## 3 Materials and methods

### 3.1 Research design

This study employs a cross-sectional survey design using quantitative methods to examine the relationships between AI tool quality dimensions, user acceptance mechanisms, and design innovation ability. The cross-sectional approach is appropriate for this exploratory research, which aims to capture a snapshot of the current relationships and test theoretical propositions about technology acceptance in AI-mediated creative contexts rather than tracking longitudinal changes. It follows a positivist epistemological stance, treating constructs as measurable variables. The approach follows the technology acceptance research tradition and enables hypothesis testing using structural equation modeling.

The study targets industrial design students in Chinese higher education institutions who have experience using AI tools in their design practice. The population was selected for three reasons:

(1) Industrial design students represent intensive users of AI tools in creative work, regularly employing generative AI, visualization tools, and parametric design systems in coursework and projects.
(2) This demographic is at a critical stage of design innovation ability development, where tool adoption patterns may significantly influence creative capability formation.
(3) The Chinese design education context provides a specific institutional and cultural setting for examining these relationships, allowing exploration of how established technology acceptance theories manifest in this particular context.

The analytical approach uses Partial Least Squares Structural Equation Modeling (PLS-SEM), selected for its suitability for exploratory research with complex mediating relationships. PLS-SEM is particularly appropriate here because it focuses on prediction and theory development rather than theory confirmation, accommodates models with multiple constructs and intricate path structures, makes minimal distributional assumptions, and performs effectively with formative or reflective measurement models.

It bears noting that the cross-sectional design captures associations at a single point in time and does not support strong causal inferences. Terms such as “influence” and “impact” used throughout this paper refer to statistically significant associations within the structural model, not to experimentally established causation. Future research employing longitudinal or experimental designs would be necessary to establish temporal precedence and causal directionality among the constructs.

### 3.2 Participants

Data collection was conducted from February to June 2025 through the online platform Wenjuanxing.^1^ The study employed a convenience sampling strategy combined with snowball recruitment. Survey links were initially distributed through discipline-specific WeChat groups and Tencent QQ groups affiliated with industrial design programs at multiple Chinese universities, covering institutions across Eastern, Central, and Western China to enhance geographic diversity. To extend reach, participants were encouraged to share the survey link with fellow industrial design students who had experience using AI tools, creating a snowball effect. Additionally, the survey was posted in the questionnaire assistance community on the Wenjuanxing platform, which connects researchers with potential respondents in relevant academic fields.

To ensure respondent eligibility, two screening questions were placed at the beginning of the questionnaire: (i) “Are you currently enrolled in an industrial design program at a Chinese university?” and (ii) “Have you used AI tools (e.g., Midjourney, DALL⋅E, ChatGPT, or similar tools) in your design coursework or projects?” Respondents who answered “No” to either question were directed to the end of the survey and excluded from the final dataset. All participants participated anonymously and voluntarily, and informed consent was obtained prior to survey completion. The research protocol received ethical exemption from the relevant Institutional Review Board (Number: CVITG/PP/24041021).

Given that the study was conducted in China, the original questionnaire was first translated from English to Chinese following a standard cross-cultural translation procedure. Two bilingual researchers independently translated the English items into Chinese, and discrepancies were resolved through discussion. Subsequently, an independent English expert performed a back-translation to ensure semantic equivalence, and minor adjustments were made where the back-translation revealed ambiguity.

A total of 1,089 questionnaires were collected. Responses were screened using the following exclusion criteria: (a) incomplete responses with more than 10% missing items, (b) responses completed in under 120 s (indicating insufficient engagement), and (c) responses exhibiting patterned answering (e.g., selecting the same option for all items). After applying these criteria, 73 invalid responses were excluded, resulting in 1,016 valid responses and an effective response rate of 93.3%. No imputation was performed, as the remaining valid responses contained no missing values. To detect potential outliers, we examined Mahalanobis distance values for the 20 measurement items; no cases exceeded the critical chi-square value at *p* < 0.001, indicating the absence of multivariate outliers.

We acknowledge that the convenience and snowball sampling approach may introduce selection bias, as students who are more active on social media or more experienced with AI tools may be overrepresented. The large sample size (*N* = 1,016) and heterogeneous distribution across academic performance levels, usage experience, and usage frequency partially mitigate this concern, though the implications for generalizability are discussed further in the Limitations section. The demographic characteristics of the participants are summarized in Table 1. Three demographic variables were collected. Academic performance was measured by self-reported major-specific ranking, categorized as Excellent (top 30% of cohort), Good (middle 40%), and Average (last 30%). This classification enables examination of potential differences in AI tool perception and usage patterns across different levels of academic performance, as higher-performing students may differ in their capacity to critically evaluate AI-generated content and their expectations regarding tool quality. Years of AI tool usage was classified into three bands: less than 2 years, 2–5 years, and more than 5 years. Frequency of AI tool usage ranged from “not commonly used” to “every day.”

**TABLE 1:** Demographic profile of participants.

Demographic profile	Category	Frequency	Percentage
Academic level	Top 30%	349	34.35%
	Middle 40%	326	32.09%
	Last 30%	341	33.56%
Years of using AI tools	Less than 2 years	358	35.24%
	2–5 years	332	32.68%
	More than 5 years	326	32.09%
Frequency of using AI tools	Not commonly used	273	26.87%
	Several times a month	252	24.80%
	Several times a week	229	22.54%
	Every day	262	25.79%

### 3.3 Measures

The analysis employed a cross-sectional design to collect data through an online survey. The questionnaire was designed following standard scale development procedures, consisting of two main sections: demographic information and measurements of the research constructs. The demographic section collected background information such as participants’ academic level, years of AI tool usage, and frequency of usage. The measurement section included five core constructs with a total of 20 measurement items. These constructs were interaction quality (IQT), information quality (INQ), satisfaction (SAT), intention to use (INU), and design innovation ability (DIA), each containing four measurement items.

All constructs were measured using a 5-point Likert scale with options ranging from 1 = “Strongly disagree” to 5 = “Strongly agree.” The measurement items were developed based on established scales and were contextualized according to the application characteristics of AI tools in design practice, ensuring that the items were relevant and applicable to the study context. The specific measurement items for each construct, along with their theoretical sources, are presented in Table 2.

**TABLE 2:** Measurement indicators and sources.

Constructs	Indicators	Sources
Interaction quality (IQT)	IQT1: AI tools can accurately understand my design needs and provide intelligent interactive feedback.	(Petter et al., 2008; Salisu et al., 2025; Luo et al., 2025)
	IQT2: AI tools can provide personalized design suggestions based on my design preferences and habits.	
	IQT3: When using AI tools, I can obtain real-time design feedback that helps improve my designs.	
	IQT4: AI tools can facilitate smooth collaboration and interaction between me and my classmates and teachers.	
Information quality (INQ)	INQ1: The design content generated by AI tools is accurate and meets professional standards.	(DeLone and McLean, 2002; Petter et al., 2008; Luo et al., 2025)
	INQ2: The design information provided by AI tools is clear and easy to understand, making it convenient for me to comprehend and apply.	
	INQ3: The design suggestions provided by AI tools are highly matched to my creative needs.	
	INQ4: AI tools provide me with rich, diverse, and high-quality design resources that enhance my design outcomes.	
Satisfaction with tools (SAT)	SAT1: I am satisfied with the AI tools I currently use.	(Zhao et al., 2012; Lam, 2025)
	SAT2: AI tools help me improve my design efficiency.	
	SAT3: I am satisfied with the functions and design features of AI tools, believing they meet my needs.	
	SAT4: Using AI tools makes me feel more confident in design creation.	
Intention to use (INU)	INU1: I am willing to use AI tools as auxiliary tools in the design process.	(Chai et al., 2024; Soliman et al., 2024)
	INU2: I am interested in trying and exploring different types of AI tools.	
	INU3: I believe learning how to use AI tools is worthwhile.	
	INU4: I intend to use AI tools for an extended period to assist with my design work.	
Design innovation ability (DIA)	DIA1: By using AI tools, I can more effectively conceive and develop innovative design concepts.	(Diedrich et al., 2018; Birdi et al., 2016; Hwang and Wu, 2025)
	DIA2: AI tools help me better plan and manage the entire creative design process.	
	DIA3: AI tools enable me to flexibly adjust design methods to adapt to the requirements of different projects.	
	DIA4: AI tools significantly enhance my design innovation ability in design solutions.	

Prior to the main data collection, a content validation procedure was conducted to evaluate the clarity, relevance, and cultural appropriateness of the translated questionnaire. Three domain experts, including two faculty members specializing in industrial design education and one researcher in information systems, independently reviewed all 20 measurement items for content relevance, wording accuracy, and alignment with the intended constructs. Additionally, five graduate students in industrial design were invited to complete the questionnaire and provide verbal feedback on item comprehensibility and cultural appropriateness. All items were judged as clear and relevant by the expert panel, and minor wording adjustments were made to two items based on their recommendations to improve clarity in the Chinese translation. No items were removed. The refined questionnaire was then used for the main study.

### 3.4 Data analysis

This study employed Partial Least Squares Structural Equation Modeling (PLS-SEM) for data analysis using SmartPLS 4.1 software. PLS-SEM was selected over covariance-based SEM (CB-SEM) for several methodological reasons aligned with the study’s objectives and characteristics. First, the research is exploratory in nature, aimed at prediction and theory development in a novel context of AI-mediated design education, rather than confirmatory testing of well-established theories. PLS-SEM is particularly well-suited for such exploratory research because it maximizes explained variance in endogenous constructs and identifies key driver variables (Hair et al., 2019). Second, the theoretical model involves multiple mediating pathways from quality through satisfaction and intention to innovation ability, and PLS-SEM provides robust estimation for models with complex indirect effects (Ali et al., 2025). Third, PLS-SEM makes minimal distributional assumptions and does not require multivariate normality, which is advantageous for Likert-scale survey data that often deviates from normality. Fourth, PLS-SEM has been successfully applied in recent studies examining technology adoption in higher education using integrated theoretical models similar to ours. For example, Soliman et al. (2023) employed PLS-SEM to model continuous intention to use learning platforms, demonstrating its effectiveness for educational technology adoption research with multiple mediating constructs. Finally, following recent methodological guidance (Hair et al., 2024), we supplemented the PLS-SEM analysis with model fit assessment indices (SRMR, NFI) to enhance the rigor of our structural model evaluation.

The sample size of 1,016 valid responses substantially exceeds the minimum requirements for PLS-SEM analysis. According to the “10 times rule,” the minimum sample size should be at least ten times the maximum number of structural paths directed at any latent variable in the model. In this study, the endogenous variable with the most predictors is intention to use (INU), which has three incoming paths (from IQT, INQ, and SAT), requiring a minimum sample of 30 cases. Our sample of 1,016 provides ample statistical power for reliable parameter estimation and hypothesis testing, allowing detection of even modest effect sizes with high power.

**FIGURE 2:**
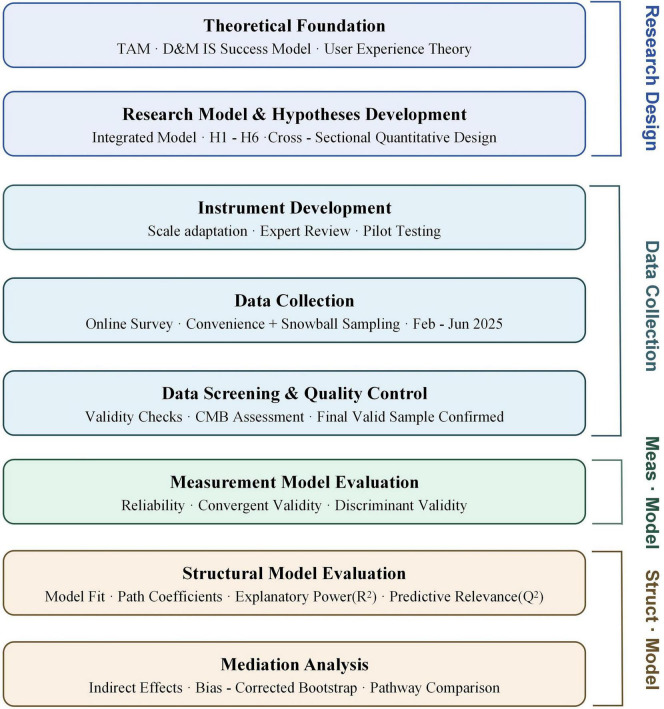
Research methodology framework.

Data analysis was conducted using SmartPLS 4.1 software, following a standard two-stage evaluation procedure. The measurement model evaluation includes internal consistency reliability tests (Cronbach’s α and composite reliability), convergent validity tests (factor loadings and Average Variance Extracted (AVE) values), and discriminant validity tests (Fornell-Larcker criterion and HTMT ratio). The structural model evaluation is performed after confirming the quality of the measurement model, including path coefficient significance testing, explanatory power assessment, and predictive relevance testing. All significance tests were conducted using a bootstrap procedure with 5,000 resamples. The predictive power of the structural model was evaluated using the coefficient of determination (R^2^) and predictive relevance (Q^2^). Figure 2 provides an overview of the full research methodology, illustrating the sequential procedures from theoretical foundation and data collection through measurement model assessment to structural model evaluation and research outputs.

## 4 Results

### 4.1 Common method bias assessment

To address potential common method variance, we employed multiple diagnostics. First, Harman’s single-factor test showed that a single factor accounted for 34.7% of total variance, below the 50% threshold (Vieira et al., 2025). However, recognizing the limitations of this traditional approach, we conducted full collinearity VIF analysis as a more robust diagnostic for PLS-SEM contexts (Hair et al., 2024). As reported in Table 3, all outer VIF values (1.957–2.417) are well below the threshold of 3.3, providing stronger evidence that common method variance does not substantially distort the structural parameter estimates. Procedural controls during data collection further minimized potential biases: all participants completed the survey anonymously through an online platform and were informed that participation was voluntary with no consequences for withdrawal.

**TABLE 3:** Full collinearity assessment results (outer model VIF values).

Indicators	VIF	Indicators	VIF	Indicators	VIF	Indicators	VIF	Indicators	VIF
IQT1	2.296	INQ1	2.065	SAT1	2.116	INU1	2.392	DIA1	2.118
IQT2	2.237	INQ2	2.077	SAT2	2.329	INU2	2.302	DIA2	2.128
IQT3	2.417	INQ3	1.957	SAT3	2.280	INU3	2.181	DIA3	2.107
IQT4	2.111	INQ4	1.969	SAT4	2.244	INU4	2.309	DIA4	2.093

Based on Harman’s single-factor test and VIF test, this study further employs the Unmeasured Latent Method Construct (ULMC) (Podsakoff et al., 2003) to conduct an additional check for common method bias. Since the ULMC test requires specific constraints on the model and compares the fit differences of nested models, and PLS-SEM, which is based on variance estimation, does not support such tests (Hair et al., 2019), this study uses covariance-based Structural Equation Modeling (CB-SEM) specifically for the ULMC test. A latent method factor connected to all observed variables was added to the original measurement model, and the model fit before and after adding the method factor was compared. As shown in Table 4, after adding the method factor, the measurement model fit improved [χ^2^(144) = 154.221 vs. χ^2^(164) = 318.891]. The ΔCFI (0.013) remained below the recommended threshold of 0.020, though the ΔRMSEA (0.022) slightly exceeded the stricter 0.015 criterion. However, three considerations support the conclusion that common method bias does not substantially distort the results. First, the method factor model achieved excellent absolute fit (CFI = 0.999, RMSEA = 0.008, SRMR = 0.016), indicating that any method variance captured by the latent factor is minor in magnitude. Second, the ΔCFI criterion is generally considered more reliable than ΔRMSEA for comparing nested measurement models, particularly in large samples where RMSEA-based differences can be overly sensitive (Cheung and Rensvold, 2002; Chen, 2007). Third, the factor loadings and inter-construct correlations in the method factor model remained consistent with those in the original measurement model, suggesting that the addition of the method factor did not substantively alter the measurement structure. Based on the convergent evidence from Harman’s single-factor test, full collinearity VIF analysis, and the ULMC test, common method bias does not pose a substantial threat to the conclusions of this study.

**TABLE 4:** Unmeasured Latent Method Construct (ULMC) test results.

Indicator	Original measurement model	Method factor model (ULMC)	Difference (Δ)	Criterion
χ^2^	318.891	154.221	164.670	−
df	164	144	20	−
χ^2^/df	1.944	1.071	−	<3.000
CFI	0.986	0.999	0.013	<0.020
RMSEA	0.030	0.008	0.022	<0.015
SRMR	0.086	0.016	0.070	−
TLI	0.984	0.999	−	−

### 4.2 Assessment of the model fit

To verify the overall goodness of fit of the model, the analysis calculated key fit indices of the PLS-SEM model using SmartPLS 4. As shown in Table 5, all fit indices meet acceptable standards: the SRMR value is 0.039 ( < 0.08), the d_ULS value is 0.313 ( < 0.95), the d_G value is 0.151 ( < 0.95), and the NFI value is 0.919 ( > 0.9) (Dijkstra and Henseler, 2015). Based on the evaluation of these fit indices, the proposed structural model has acceptable fit, meeting the prerequisites for further structural model analysis.

**TABLE 5:** Model fit assessment results.

Indicators	Judgment criteria (threshold)	Value
SRMR	<0.08	0.039
d_ULS	<0.95	0.313
d_G	<0.95	0.151
Chi-square	−	912.867
NFI	>0.9	0.919

### 4.3 Measurement model evaluation

According to the standard PLS-SEM analysis process, the measurement model evaluation includes three key steps: internal consistency reliability, convergent validity, and discriminant validity (Hair et al., 2019). Internal Consistency Reliability was assessed using Cronbach’s α, composite reliability (CR), and the Dijkstra-Henseler rho_A (ρA). The acceptable threshold for these three indices is 0.70. As shown in Table 6, the Cronbach’s α values for all constructs range from 0.862 to 0.886, the CR values range from 0.906 to 0.921, and the ρA values range from 0.864 to 0.887. All values exceed the recommended threshold, indicating good internal consistency reliability for each construct.

**TABLE 6:** Internal consistency reliability assessment.

Constructs	Cronbach’s alpha	Composite reliability (CR)	Rho_A
IQT	0.883	0.920	0.884
INQ	0.862	0.906	0.864
SAT	0.871	0.912	0.874
INU	0.881	0.918	0.882
DIA	0.886	0.921	0.887

Convergent Validity was assessed using factor loadings and Average Variance Extracted (AVE). The evaluation criteria for these were factor loadings greater than 0.70 and AVE greater than 0.50 (Hair et al., 2019). As shown in Table 7, the factor loadings for all indicators range from 0.831 to 0.873, and the AVE values for each construct range from 0.708 to 0.745, all of which meet the recommended criteria. These results indicate that the measurement model has good convergent validity.

**TABLE 7:** Convergent validity assessment results.

Constructs	Indicators	Factor loadings	Average variance extracted (AVE)
IQT	IQT1	0.866	0.741
	IQT2	0.857	
	IQT3	0.873	
	IQT4	0.846	
INQ	INQ1	0.849	0.708
	INQ2	0.842	
	INQ3	0.831	
	INQ4	0.842	
SAT	SAT1	0.846	0.721
	SAT2	0.866	
	SAT3	0.863	
	SAT4	0.860	
INU	INU1	0.847	0.738
	INU2	0.859	
	INU3	0.843	
	INU4	0.848	
DIA	DIA1	0.867	0.745
	DIA2	0.859	
	DIA3	0.859	
	DIA4	0.866	

Discriminant validity tests aim to ensure statistical differentiation between different constructs. The study employed two complementary methods to test this: the Fornell-Larcker criterion and the Heterotrait-Monotrait (HTMT) ratio. The Fornell-Larcker criterion requires that the square root of each construct’s AVE should be greater than its correlation with other constructs (Henseler et al., 2015). As shown in Table 8, the square roots of the AVE values for all constructs (diagonal values: 0.841–0.863) are greater than the correlations with other constructs (ranging from 0.270 to 0.424), fulfilling the requirements for discriminant validity.

**TABLE 8:** Discriminant validity assessment (Fornell-Larcker criterion).

Constructs	DIA	INQ	INU	IQT	SAT
DIA	0.863*	**–**	**–**	**–**	**–**
INQ	0.410	0.841*	**–**	**–**	**–**
INU	0.424	0.285	0.859*	**–**	**–**
IQT	0.413	0.306	0.270	0.861*	**–**
SAT	0.423	0.313	0.299	0.298	0.849*

Bold values on the diagonal indicate the square root of Average Variance Extracted (AVE).

*p < 0.05.

To further confirm discriminant validity, the HTMT ratio method was also applied. Table 9 shows that all HTMT values between constructs range from 0.306 to 0.483, all of which are below the conservative threshold of 0.85 (or 0.90 for a more liberal threshold). The highest HTMT value is 0.483 (between DIA and SAT), which is still below the discriminant validity threshold. This result further confirms that the constructs are statistically distinct from one another.

**TABLE 9:** Discriminant validity assessment (HTMT ratio).

Constructs	DIA	INQ	INU	IQT	SAT
DIA	−	−	−	−	−
INQ	0.468				
INU	0.479	0.326			
IQT	0.467	0.350	0.306		
SAT	0.483	0.359	0.341	0.338	

The consistency of the results from the two testing methods enhances the reliability of the discriminant validity assessment.

### 4.4 Structural model evaluation

After confirming the suitability of the measurement model, this study evaluates the structural model to verify the theoretical relationship hypotheses. The structural model evaluation follows the standard PLS-SEM procedure, including collinearity diagnostics, path coefficient tests, explanatory power assessments, and predictive relevance evaluations (Hair et al., 2019). First, collinearity diagnostics are performed to ensure the validity of the regression results. Collinearity among latent variables is assessed using Variance Inflation Factors (VIF), with the evaluation standard set to a VIF value lower than 5.0. As shown in Table 10, the internal VIF values of all paths range from 1.000 to 1.173, all below the critical threshold, indicating that collinearity does not affect the structural model estimation.

**TABLE 10:** Collinearity assessment (Inner VIF values).

Path	VIF value	Assessment
INQ → INU	1.173	No collinearity
INQ → SAT	1.103	No collinearity
INU → DIA	1.000	No collinearity
IQT → INU	1.161	No collinearity
IQT → SAT	1.103	No collinearity
SAT → INU	1.167	No collinearity

**FIGURE 3:**
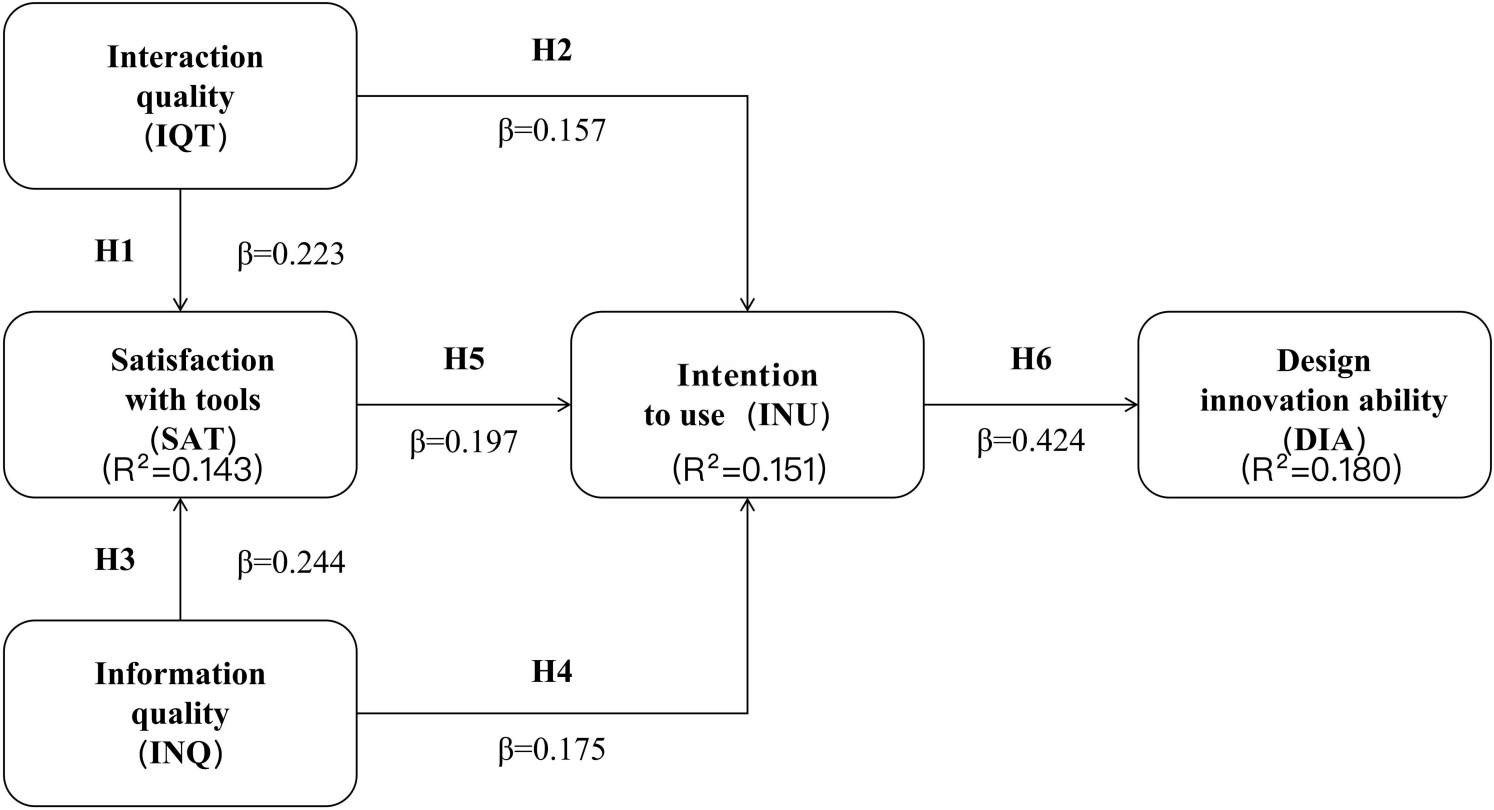
Structural model of AI tools’ impact on users’ design innovation ability.

The analysis employed 5,000 bootstrap resamples for path coefficient significance tests (Streukens and Leroi-Werelds, 2016). As shown in Figure 3 and Table 11, all six research hypotheses are supported (*p* < 0.001). Specifically, IQT has a significant positive effect on SAT (β = 0.223, *t* = 7.039, *f*^2^ = 0.042) and INU (β = 0.157, *t* = 5.000, *f*^2^ = 0.088), supporting H1 and H2; INQ has a significant positive effect on SAT (β = 0.244, *t* = 8.016, *f*^2^ = 0.051) and INU (β = 0.175, *t* = 5.989, *f*^2^ = 0.081), supporting H3 and H4; SAT has a significant positive effect on INU (β = 0.197, *t* = 6.070, *f*^2^ = 0.105), supporting H5; INU has a significant positive effect on DIA (β = 0.424, *t* = 16.922, *f*^2^ = 0.220), supporting H6. Following Cohen’s (1988) benchmarks, most paths exhibit small effect sizes (*f*^2^ = 0.042 to 0.105), while the INU → DIA path demonstrates a medium effect size (*f*^2^ = 0.220), indicating that intention to use makes the largest unique explanatory contribution among all structural relationships in the model.

**TABLE 11:** Structural model examination results.

Hypotheses	Paths	Standardized path coefficients (β)	*f* ^2^	T-statistics	*P*-values	Remarks
H1	IQT → SAT	0.223	0.042	7.039	<0.001	Supported
H2	IQT → INU	0.157	0.088	5.000	<0.001	Supported
H3	INQ → SAT	0.244	0.051	8.016	<0.001	Supported
H4	INQ → INU	0.175	0.081	5.989	<0.001	Supported
H5	SAT → INU	0.197	0.105	6.070	<0.001	Supported
H6	INU → DIA	0.424	0.220	16.922	<0.001	Supported

*f*^2^ effect sizes are interpreted following Cohen (1988): 0.02, small; 0.15, medium; 0.35, large.

R-squared (R^2^) is used to assess the explanatory power of the structural model for endogenous constructs. The evaluation standards are 0.75, 0.50, and 0.25, which represent strong, medium, and weak explanatory power, respectively. As shown in Table 12, IQT and INQ together explain 14.3% of the variance in SAT (*R*^2^ = 0.143), IQT, INQ, and SAT together explain 15.1% of the variance in INU (*R*^2^ = 0.151), and INU explains 18.0% of the variance in DIA (*R*^2^ = 0.180). The explanatory power for each endogenous construct ranges from weak to medium, but all *R*^2^-values are statistically significant (*p* < 0.001), indicating that the model is statistically meaningful.

**TABLE 12:** R-square assessment results.

Endogenous construct	*R* ^2^	Adjusted *R*^2^	Effect size
SAT	0.143	0.141	Small
INU	0.151	0.148	Small
DIA	0.180	0.179	Small-Medium

The structural model results show that IQT (β = 0.223) and INQ (β = 0.244) have significant positive effects on SAT, explaining 14.3% of the variance; IQT (β = 0.157), INQ (β = 0.175), and SAT (β = 0.197) have significant positive effects on INU, explaining 15.1% of the variance; INU (β = 0.424) has a significant positive effect on DIA, explaining 18.0% of the variance (all *p* < 0.001). Predictive relevance (Q^2^) is evaluated using the PLSpredict algorithm, assessing the model’s out-of-sample prediction accuracy. A Q^2^ value greater than 0 indicates that the model has predictive relevance. As shown in Table 13, all endogenous constructs have positive Q^2^ values: SAT (Q^2^ = 0.138), INU (Q^2^ = 0.113), and DIA (Q^2^ = 0.126), indicating that the model has acceptable predictive power.

**TABLE 13:** Predictive relevance assessment results.

Endogenous construct	*Q* ^2^
SAT	0.138
INU	0.113
DIA	0.126

In conclusion, the structural model evaluation results indicate that the theoretical model constructed in this study is statistically significant and theoretically valuable. Although the explanatory power of the model is weak to medium (R^2^ ranging from 0.143 to 0.180), all hypothesis paths are statistically significant, and the predictive relevance indicators (Q^2^) are positive, confirming the model’s predictive validity. Considering the complexity in behavioral science research, this level of explanatory power is acceptable for exploratory research.

### 4.5 Inspecting the mediating effects

Based on the theoretical model assumptions, satisfaction is set as the mediating variable connecting the quality features of AI tools (interaction quality and information quality) with the intention to use. According to the Technology Acceptance Model and Expectation Confirmation Theory, the user’s perception of technological features indirectly influences behavioral intentions through satisfaction. While the structural model results show that both interaction quality and information quality have significant direct effects on the intention to use, the theoretical framework suggests that satisfaction may play a mediating role in these relationships.

The research employed PLS-SEM to test the mediating effects, using the bias-corrected bootstrap method with 5,000 resamples to compute the confidence intervals for the indirect effects (Li and Zhu, 2022). The criteria for judging mediating effects are as follows: if both the indirect and direct effects are significant, it is considered partial mediation; if only the indirect effect is significant but the direct effect is not, it is considered full mediation.

Table 14 presents the mediation analysis results. Two significant mediating paths were identified, both exhibiting complementary partial mediation. Satisfaction (SAT) partially mediates the effect of interaction quality (IQT) on intention to use (INU), with an indirect effect of 0.044 (*t* = 4.591, *p* < 0.01) and a direct effect of 0.157 (*t* = 5.000, *p* < 0.001). Similarly, satisfaction (SAT) partially mediates the effect of information quality (INQ) on intention to use (INU), with an indirect effect of 0.048 (*t* = 4.754, *p* < 0.01) and a direct effect of 0.175 (*t* = 5.989, *p* < 0.001). In both paths, the direct effects substantially exceed the indirect effects, indicating that while satisfaction serves as a significant supplementary channel, quality perceptions predominantly influence usage intentions through direct pathways. These results support the hypothesized mediating role of satisfaction in the quality–intention relationship.

**TABLE 14:** Mediation analysis results.

Paths	Indirect effects	*T*-values	Direct effects	*T*-values	Remarks
IQT → SAT → INU	0.044**	4.591	0.157***	5.000	Complementary partial mediation
INQ → SAT → INU	0.048**	4.754	0.175***	5.989	Complementary partial mediation

**p < 0.01,

***p < 0.001.

## 5 Discussion

The structural model results confirmed all six hypothesized relationships, supporting the integrated theoretical framework linking AI tool quality dimensions to design innovation ability through user acceptance mechanisms. However, the primary theoretical contribution resides not in this directional confirmation, which is broadly consistent with established TAM and D&M predictions, but in unexpected patterns regarding the relative strength and boundary conditions of these relationships. The following sections discuss these patterns in turn.

### 5.1 Re-weighting AI tool quality dimensions in creative education

A consistent pattern across all outcome variables is that information quality exerts stronger effects than interaction quality on both satisfaction (β = 0.244 vs. 0.223) and intention to use (β = 0.175 vs. 0.157). While these differences are numerically modest, their consistency across both dependent variables suggests a systematic rather than chance pattern. This pattern is largely corroborated by f^2^ effect sizes. For the satisfaction path, information quality (*f*^2^ = 0.051) shows a larger explanatory contribution than interaction quality (*f*^2^ = 0.042). For intention to use, the *f*^2^-values are comparable (IQT: *f*^2^ = 0.088; INQ: *f*^2^ = 0.081), with interaction quality showing a marginally higher effect size despite a slightly lower path coefficient. This minor divergence likely reflects the shared variance structure among predictors. Overall, all individual *f*^2^-values fall within the small effect range (Cohen, 1988), suggesting that both quality dimensions make meaningful but modest unique contributions to explaining user responses.

This finding challenges a widespread assumption in technology acceptance research. Classical TAM scholarship has established that perceived ease of use, the construct most analogous to interaction quality, is the primary driver of early adoption, with perceived usefulness gaining importance only as users accumulate experience (Davis, 1989; Venkatesh and Bala, 2008). Following this logic, educators and tool developers have prioritized interface usability as the gateway to engagement. Our results reveal a reversed priority in generative AI contexts. Design students appear to evaluate AI tools primarily as content generators rather than as interfaces, privileging the quality of AI-produced design alternatives over the smoothness of the interaction process.

This re-weighting can be understood through the lens of Cognitive Load Theory (CLT) (Sweller, 1988). In design tasks, the primary cognitive challenge is not navigating an interface (extraneous load) but evaluating and integrating generated content into creative solutions (germane load). When AI tools produce high-quality, relevant, and diverse design outputs, they directly reduce the germane cognitive load associated with ideation, enabling students to allocate cognitive resources to higher-order creative synthesis. By contrast, interaction quality primarily reduces extraneous load, a benefit that, while welcome, is secondary to the core creative task. This cognitive load interpretation aligns with Ahmad et al. (2025), who found that content quality was the strongest predictor of AI tool effectiveness in knowledge work, and extends their findings to creative education contexts.

The finding also converges with recent AI adoption research. Soliman et al. (2024) reported that performance expectancy, closely related to our information quality construct, was the strongest predictor of continued intention to use generative AI among university students, outperforming effort expectancy (analogous to interaction quality). Similarly, Luo et al. (2025) found information quality to surpass system quality in predicting satisfaction with AI-powered educational tools. Our results add to this emerging evidence base by demonstrating the pattern specifically in creative education contexts, where the content-generative nature of AI tools makes output quality particularly salient.

However, this finding diverges from studies in consumer AI applications. Li (2023) found that interaction quality dominated satisfaction formation for AI chatbots in general educational use, suggesting that the re-weighting we observe may be specific to creative domains where output quality directly determines task outcomes. This divergence underscores the importance of context-sensitive theorizing about AI tool acceptance rather than assuming universal mechanisms.

### 5.2 The new balance between affective and cognitive pathways

The second notable pattern concerns the relative strength of mediated versus direct pathways. Both interaction quality and information quality exert direct effects on intention to use that are approximately three to four times stronger than their indirect effects through satisfaction (direct: β = 0.157 and 0.175; indirect: β = 0.044 and 0.048). This pattern indicates that in this educational context, cognitive-functional evaluation substantially dominates affective-experiential evaluation as a driver of usage decisions. The f^2^ statistics provide further evidence for this interpretation. Satisfaction’s effect on intention to use yields a small to medium f^2^ of 0.105, indicating a meaningful but limited unique explanatory contribution as a mediator. By contrast, the direct effects of interaction quality (*f*^2^ = 0.088) and information quality (*f*^2^ = 0.081) on intention to use are of comparable magnitude, suggesting that the cognitive-functional pathway operates with a strength similar to the affective pathway at the individual predictor level. Notably, the strongest f^2^ in the entire model belongs to the INU → DIA path (*f*^2^ = 0.220, medium effect), confirming that the adoption to capability link represents the most substantively important relationship in the framework.

This finding departs from the predictions of Expectation Confirmation Theory (Oliver, 1980) and affective technology acceptance models, which position satisfaction as a central mediator in the quality-to-intention relationship. In consumer technology contexts, satisfaction typically plays a dominant mediating role because adoption decisions are heavily influenced by hedonic responses (Han et al., 2024; Nafees and Sujood, 2025). The limited mediation we observe suggests that design students operate in a qualitatively different evaluative mode.

Self-Determination Theory (SDT) (Ryan and Deci, 2000) offers a useful interpretive framework. SDT distinguishes between intrinsic motivation (driven by enjoyment and satisfaction) and extrinsic motivation (driven by instrumental outcomes). In Chinese design education, where assessment is heavily outcome-oriented and students face institutional pressure to demonstrate tangible portfolio achievements, extrinsic motivation may systematically outweigh intrinsic motivation in tool evaluation. Students assess AI tools primarily for their competence-enhancing utility, whether the tool helps produce better designs, rather than for the satisfaction derived from the interaction experience. This interpretation is consistent with the autonomy-competence-relatedness framework of SDT: when the competence dimension dominates (producing good work), hedonic satisfaction becomes secondary.

The practical implication is significant. Conventional user experience design principles, derived largely from consumer product contexts, emphasize that positive emotional experiences drive continued engagement. Our finding suggests that this principle may not transfer directly to educational tools used for creative work. Design students appear to tolerate imperfect interaction experiences if the tool delivers substantive creative value. This does not mean satisfaction is irrelevant, it remains a statistically significant partial mediator, but its role is substantially diminished relative to direct functional evaluation.

The finding also has methodological implications for future research. Studies using satisfaction as the sole mediator between quality and intention may underestimate the total effect of quality perceptions in educational contexts, as the direct pathway captures evaluation processes that bypass affective mediation.

### 5.3 The adoption-to-ability link: strong effect, substantial unexplained variance

The relationship between intention to use and design innovation ability (β = 0.424, *p* < 0.001) represents the strongest path in our model and confirms the extension of technology acceptance frameworks beyond adoption behavior to educational outcomes. This effect size is comparable to or exceeds intention-behavior relationships reported in classic TAM meta-analyses (Venkatesh and Bala, 2008), providing evidence that AI tool adoption motivation does translate meaningfully into creative ability development.

Yet the explained variance is modest (*R*^2^ = 0.180), meaning that 82% of the variation in design innovation ability remains outside the scope of technology acceptance constructs. This coexistence of a strong path coefficient with limited explanatory power constitutes what we term the “design innovation ability paradox,” and it is arguably the central finding of this study.

The paradox carries three theoretical implications. First, it validates the adoption-to-ability extension: technology acceptance is a meaningful, if incomplete, predictor of educational outcomes, bridging the gap between IS research (which stops at adoption) and educational research (which focuses on outcomes). Second, it reveals the boundary of technology acceptance theory in educational contexts. The 82% unexplained variance is not a sign of model failure, our model demonstrates acceptable fit and measurement quality, but rather an indicator of theoretical incompleteness. Design innovation ability development is a complex process shaped by multiple factors beyond technology acceptance. These factors include pedagogical design, such as how AI tools are integrated into curricula and assignments, as well as instructor scaffolding in the form of guidance on effective AI collaboration strategies. Individual differences also play a role, encompassing prior creative ability, domain expertise, metacognitive skills, and AI literacy. Additionally, peer interaction through collaborative critique and discussion of AI-generated outputs, along with motivational factors such as creative self-efficacy and growth mindset, may further influence this developmental process.

Third, the paradox carries a critical pedagogical message: promoting AI tool adoption is necessary but insufficient for design innovation ability development. Educators cannot assume that merely increasing students’ willingness to use AI tools will automatically enhance their creative capabilities. Rather, the substantial unexplained variance suggests that how students use AI tools, the quality and depth of their engagement, the pedagogical scaffolding surrounding tool use, and the development of critical evaluation skills, matters as much as, or more than, whether they intend to use them.

This finding also warrants critical reflection through the lens of cognitive offloading theory (Risko and Gilbert, 2016). While our aggregate results support the technology enhancement perspective (higher intention predicts higher design innovation ability), the modest R^2^ may mask heterogeneity in the relationship. Some students may develop genuine creative capabilities through deep, critical engagement with AI tools, while others may develop dependency patterns that inflate self-reported design innovation ability without corresponding ability gains. This interpretation aligns with the self-report measurement concern raised for DIA, self-reported design innovation ability may partly reflect confidence and familiarity rather than actual creative ability. Future research employing objective creativity measures alongside self-report instruments, or using person-centered analytic approaches such as latent profile analysis, would help disentangle these possibilities.

### 5.4 Contextual interpretation

The patterns discussed in sections 5.1–5.3 should be interpreted with attention to the specific institutional and cultural context of Chinese design education, while avoiding overgeneralized cultural attributions.

At the institutional level, several concrete features of the Chinese design education system may shape the observed patterns. Chinese design programs typically employ structured curricula with clearly defined assessment rubrics that evaluate tangible design outputs (portfolios, prototypes, competition submissions) rather than process-oriented measures such as creative exploration or experimental risk-taking (Zhan et al., 2022). This outcome-focused assessment structure creates an evaluative environment in which students rationally prioritize tools that demonstrably improve output quality over tools that provide enjoyable interaction experiences. The institutional emphasis on employment preparation and portfolio competitiveness further reinforces this instrumental orientation.

At the pedagogical level, the integration of AI tools in Chinese design curricula is still in an early, largely instructor-directed phase. Many programs introduce AI tools through structured assignments with specific output requirements rather than through open-ended exploration. This structured integration may channel students’ attention toward evaluating whether AI-generated content meets assignment specifications (information quality) rather than toward appreciating the fluidity of the creative interaction process (interaction quality).

At the individual level, the modest R^2^ for design innovation ability may partly reflect the measurement challenge inherent in self-reported creativity assessment. Chinese students, socialized in an educational culture that values humility and external validation over self-promotion, may underreport creative capabilities or may calibrate their self-assessments differently than students in educational cultures that explicitly encourage creative self-expression. These measurement-culture interactions represent a methodological factor that should be considered alongside substantive interpretations.

These contextual factors provide more specific, empirically grounded explanations than broad cultural attributions to “Confucian traditions” or “utilitarian education.” We explicitly avoid framing these findings as evidence of fixed cultural characteristics and instead interpret them as consequences of specific institutional structures and pedagogical practices that can evolve over time and vary across institutions within China.

### 5.5 Implications

#### 5.5.1 Theoretical implications

This study contributes to three areas of theoretical development. First, it provides empirical evidence for domain-specific calibration of quality dimension weights in technology acceptance models. Rather than assuming usability universally drives adoption, the findings demonstrate that the primary value driver shifts toward content generation quality when tools function as creative collaborators, a distinction with implications for how acceptance models are specified in emerging AI contexts.

Second, it establishes boundary conditions for satisfaction-mediated acceptance models. The substantially weaker indirect pathways observed here indicate that affective mediation, while statistically present, cannot be assumed to play the central role attributed to it in consumer-derived frameworks when the evaluative context is outcome-oriented professional education.

Third, by extending the dependent variable from adoption to ability development and quantifying the resulting explanatory gap, the study opens an integrative research agenda. The substantial unexplained variance in design innovation ability provides empirical grounds for future models that combine technology acceptance constructs with pedagogical, social-cognitive, and individual difference variables.

#### 5.5.2 Practical implications

The information quality dominance finding provides clear design priorities. When developing AI tools for creative education, investment in content generation algorithms, improving the accuracy, relevance, diversity, and novelty of AI-generated design outputs, will yield greater adoption and ability development impact than equivalent investment in interface refinement. This does not mean interaction quality can be neglected, but rather that development resources should be weighted toward output quality as the primary value driver.

Three specific strategies emerge from our findings. First, tool selection criteria should prioritize content generation quality. When choosing AI tools for coursework, educators should evaluate tools based on the quality, diversity, and domain relevance of their outputs rather than on interface aesthetics or interaction fluency. Second, assignment design should promote critical engagement rather than passive consumption. Assignments requiring students to generate multiple AI-produced alternatives, critically evaluate each against design criteria, and synthesize selected elements with original concepts can build the intentional, deep engagement that our findings link to development. Third, scaffolding should balance AI assistance with cognitive independence. Specific strategies include requiring initial manual sketching before AI exploration (preserving independent ideation), structured reflection journals documenting what students learned from AI outputs versus their own contributions, and peer critique sessions where students must justify AI-assisted design decisions, practices that address the cognitive offloading risk while leveraging AI’s generative capabilities.

China’s national AI education initiatives would benefit from evidence-based guidelines on AI tool integration in creative programs. Our findings suggest that policies should move beyond encouraging AI tool adoption toward specifying quality standards for AI-generated educational content and requiring pedagogical training for instructors on effective AI integration strategies. The adoption-ability gap we identify indicates that policy success should be measured not by adoption rates but by demonstrable ability development outcomes.

## 6 Conclusion

This study demonstrates that classical technology acceptance mechanisms require systematic recalibration when applied to generative AI tools in creative education. By integrating TAM, the D&M IS Success Model, and User Experience Theory into a unified framework and testing it with 1,016 Chinese industrial design students, the study reveals that the quality–acceptance–ability chain operates through mechanisms that diverge from conventional predictions: content quality outweighs interface usability as the primary driver, direct functional evaluation outweighs affective mediation, and adoption motivation constitutes a necessary but insufficient condition for ability development.

These divergences collectively suggest that generative AI tools represent a qualitatively distinct category within technology acceptance research, one that demands context-sensitive theorizing rather than direct extrapolation from prior frameworks. For educators and policymakers, the central message is that promoting AI tool adoption must be accompanied by deliberate pedagogical design that cultivates critical, deep engagement with AI-generated content. These findings, grounded in the specific context of Chinese design higher education, invite cross-cultural replication and longitudinal extension to establish the generalizability and temporal stability of the observed patterns.

## 7 Limitations and future research

This study has several limitations that should be considered when interpreting the findings and that simultaneously suggest productive directions for future investigation.

The most significant limitation concerns the geographical and cultural specificity of the sample. The study was conducted exclusively among Chinese industrial design students recruited through convenience and snowball sampling via social media platforms. The observed patterns, information quality dominance, cognitive pathway predominance, and the adoption-ability gap, may be shaped by contextual factors specific to the Chinese educational system, including outcome-focused assessment structures, institutional pressure for portfolio competitiveness, and policy-driven AI adoption. The findings therefore cannot be directly generalized to other cultural contexts. Future research should conduct cross-country comparative studies, particularly contrasting the Chinese context with Western educational systems (where experiential learning and intrinsic motivation may be emphasized differently), with educational systems in developing countries (where institutional support for AI varies), and with professional practice settings outside academia. Such comparisons would help distinguish universal AI tool acceptance mechanisms from culturally specific manifestations. Additionally, stratified sampling based on institutional type (research universities vs. applied colleges), geographic region, and program ranking would strengthen the representativeness of findings within the Chinese context. Specifically, future research could adopt stratified random sampling to include design institutions from eastern, central, and western regions of China and employ multi-group analysis to examine the stability of the model across different institutional types.

The cross-sectional design captures associations at a single time point and cannot establish temporal precedence or causal directionality among constructs. Terms such as “influence” and “impact” in this paper refer to statistical associations, not experimentally demonstrated causation. Future research should employ longitudinal designs tracking the same cohort of students over multiple semesters to examine how AI tool quality perceptions, satisfaction, intention to use, and design innovation ability co-evolve over time. Experimental or quasi-experimental designs, for example randomly assigning students to AI tools with systematically varied interaction quality and information quality levels, would provide stronger evidence for causal claims. In particular, a three-wave longitudinal design (e.g., beginning, middle, and end of the semester) using cross-lagged panel models would enable testing of causal directionality between AI tool usage patterns and design innovation ability development over time.

Design innovation ability was measured entirely through self-report scales, which may capture self-confidence and familiarity rather than actual creative ability. This measurement approach, while common in large-scale survey research, represents a construct validity concern for a variable intended to reflect ability development. Future research should integrate objective creativity assessments, such as expert evaluations of design portfolios using the Consensual Assessment Technique (Amabile, 1982), with self-report measures to achieve methodological triangulation of the design innovation ability construct. Standardized creativity tests, such as the Torrance Tests of Creative Thinking (Torrance, 1974), could further complement these approaches. Furthermore, the absence of a formal pilot study with quantitative reliability testing prior to main data collection is acknowledged as a limitation; future studies should conduct full-scale pilot studies to establish preliminary psychometric properties.

The study relied exclusively on quantitative survey data, which captures breadth but not depth of understanding. The substantial unexplained variance in design innovation ability (82%) suggests that qualitative factors including pedagogical strategies, instructor scaffolding approaches, individual creative processes, and peer interaction dynamics play pivotal roles that cannot be captured through closed-ended survey items. Future research should adopt mixed-methods designs, combining survey data with qualitative approaches such as semi-structured interviews, classroom observation, and think-aloud protocols during AI-assisted design tasks. Such approaches could illuminate the black box between intention to use and design innovation ability development that our quantitative model identifies but cannot explain.

The study treated AI tools as a homogeneous category without distinguishing among generative image tools (e.g., Midjourney, DALL⋅E), conversational AI assistants (e.g., ChatGPT), and parametric design systems (e.g., Grasshopper with AI plugins). These tool types likely involve different interaction modalities, quality dimensions, and educational impacts. Future research should employ tool-type-specific models or multi-group analysis comparing acceptance mechanisms across different AI tool categories. For instance, future investigations could construct subgroup models distinguishing among image-generation tools (e.g., Midjourney), conversational AI systems (e.g., ChatGPT), and parametric design platforms (e.g., Grasshopper with AI plugins), and conduct multi-group comparisons to identify tool-type-specific acceptance mechanisms. Such differentiation would yield more precise guidance for tool selection and curriculum design.

Although the sample exhibited demographic diversity, the present analysis did not systematically examine whether the structural model operates differently across student subgroups. Future research should conduct multi-group analyses comparing model parameters across academic levels (undergraduate vs. graduate), AI usage experience levels (novice vs. experienced), gender, and institutional types to identify potential moderating effects. Person-centered approaches such as latent profile analysis could identify distinct patterns of AI tool engagement (e.g., deep critical users vs. passive dependent users) and their differential associations with design innovation ability.

The modest explanatory power for design innovation ability indicates that technology acceptance constructs alone are insufficient. Future research should develop integrated models that incorporate pedagogical variables (instructional design, assessment methods, AI-specific teaching strategies), social-cognitive variables (creative self-efficacy, AI literacy, growth mindset), and contextual variables (institutional support, peer collaboration norms) alongside technology acceptance constructs. Such integrated frameworks could substantially advance the prediction and understanding of AI-assisted design innovation ability development in design education.

## Data Availability

The raw data supporting the conclusions of this article will be made available by the authors, without undue reservation.

## Footnotes

1
www.wjx.cn
